# "They all work...when you stick to them": A qualitative investigation of dieting, weight loss, and physical exercise, in obese individuals

**DOI:** 10.1186/1475-2891-7-34

**Published:** 2008-11-24

**Authors:** Samantha L Thomas, Jim Hyde, Asuntha Karunaratne, Rick Kausman, Paul A Komesaroff

**Affiliations:** 1 Centre for Ethics in Medicine in Society, Department of Medicine, Monash University, Commercial Road, Prahran, Victoria 3181, Australia; 2 Department of Human Services, Victoria, Australia; 3 Butterfly Foundation and Deakin University, Melbourne, Australia

## Abstract

**Background:**

To explore the extent to which people living with obesity have attempted to lose weight, their attitudes towards dieting, physical exercise and weight loss solutions, why their weight loss attempts have failed, and their opinions about what would be most beneficial to them in their struggle with their weight.

**Method:**

Qualitative study, using open-ended interviews, of 76 people living with obesity in Victoria, Australia in 2006/7. Individuals with a BMI of 30 or over were recruited using articles in local newspapers, convenience sampling, and at a later stage purposive sampling techniques to diversify the sample. Data analysis was conducted by hand using a constant, comparative method to develop and test analytical categories. Data were interpreted both within team meetings and through providing research participants the chance to comment on the study findings.

**Results:**

Whilst participants repeatedly turned to commercial diets in their weight loss attempts, few had used, or were motivated to participate in physical activity. Friends or family members had introduced most individuals to weight loss techniques. Those who took part in interventions with members of their social network were more likely to report feeling accepted and supported. Participants blamed themselves for being unable to maintain their weight loss or 'stick' to diets. Whilst diets did not result in sustained weight loss, two thirds of participants felt that dieting was an effective way to lose weight.

**Conclusion:**

Individuals with obesity receive numerous instructions about what to do to address their weight, but very few are given appropriate long term guidance or support with which to follow through those instructions. Understanding the positive role of social networks may be particularly important in engaging individuals in physical activity. Public health approaches to obesity must engage and consult with those currently living with obesity, if patterns of social change are to occur.

## Background

Public perception, even that of some health professionals, is that people who are classified as obese are lazy and have made few serious attempts to lose weight [1]. We are now starting to understand that the underlying causes of the obesity epidemic operate at numerous levels – individual, cultural, societal, and biological. We also acknowledge that fad diets are not the solution to weight loss [2], and that we need to consider a number of short and long term strategies to facilitate the social change needed to enable individuals, families and communities to live healthy lifestyles [3].

Whilst interventions based around physical activity and behavioural therapy for those living with obesity have shown to have positive impacts on wellbeing [4,5], many obese individuals still rely on 'quick fix' strategies in their ongoing and often life-long, struggle with their weight [6]. Furthermore, physicians still recommend many popular commercial diets as individual strategies for weight loss, even though they have been shown not to be effective. Why then are diets still so seductive for obese individuals? Whilst some studies have attempted to quantify the different diets that obese people have been on [7,8], and the motivations for weight loss attempts [9], few studies have qualitatively explored the underlying motivating factors of obese people's weight loss attempts, their beliefs and expectations of diets, and the long and short term physical and emotional health effects of weight loss attempts, and what they themselves think are the optimal solutions in helping them manage their weight [10].

The results reported in this paper were part of a larger study looking at the health and social experiences of people living with obesity in the state of Victoria, Australia [11,12]. The aim of this paper was to explore in detail the extent to which people living with obesity have attempted to lose weight, their attitudes towards dieting and weight loss solutions, their opinions about why their weight loss attempts have failed, and suggestions as to what may help them in their struggle with their weight.

## Methods

The study based in Victoria, Australia, aimed to develop a picture of both lived experiences of obesity and the impact of socio-cultural factors on obesity. Given the limited research documenting the narratives of obese individuals, a qualitative research design was utilised. Qualitative studies do not aim to provide data that is statistically reliable, but instead aim to give deep insights into the experiences of individuals, in their own words. The quotes and data presented in qualitative studies aim to be illustrative of the many diverse experiences of a group of individuals and the points at which any similarities and differences occur. The study did not seek to be generalisable, but aimed to provide insights into the experiences that some obese individuals have in their constant weight loss attempts.

A broad, open-ended interview schedule was developed based on an extensive literature review, consultation with public health experts, and discussions with obese individuals. Topics included participants' experiences of commercial diets; how they had engaged in particular dieting strategies; whether diets had worked for them; the impact of dieting on their physical and emotional health; and wellbeing; and their attitudes towards physical activity. We used multiple recruitment strategies to reach as broad a range of individuals as possible. These included articles in local newspapers, convenience and snowball sampling techniques, and at a later stage purposive sampling to diversify the sample to include men, individuals under 25 and those living in rural and remote areas. Individuals were reimbursed for any travel expenses that they incurred. Interviews were conducted between September and October 2006 and were audio-taped. Participants were able to choose if they would like to be interviewed face to face, or by telephone. The latter was extremely important, as it allowed us to include a) individuals who were embarrassed or reluctant to meet with the researchers in person, b) individuals who were not mobile enough to attend a face to face interview, and c) those who lived in remote or rural locations. Interviews lasted between 60 and 120 minutes.

We used a constant, continuous, comparative method to develop analytical categories, test our processes of analysis, and then provide an explanation of why categories occurred [13]. Initial analysis was carried out by one of the study authors (ST). Each of the transcripts were read and re-read. Notes were made about the thematic and conceptual categories that emerged, why these categories were emerging, and areas of similarities and differences. A process of inter-rater reliability was undertaken [14]. A random number of interviews were selected and analysed by other members of the team to check for validity in the interpretation of data. Where there were differences, these were discussed in detail until consensus was reached. Once we were satisfied that no new themes were emerging (analytical saturation), recruitment and interviewing were stopped. After the analysis was completed, participants were sent a copy of the findings and were encouraged to contact the study team with any comments or thoughts.

## Results

One hundred and one individuals enquired about the study. Of those, 17 people refused participation after receiving further information about the study. Eight individuals did not turn up for the interview. The study demographics are presented in Additional file 1. A total of 76 individuals participated in the study. Participants were aged between 16 and 72 years old (average 47 years), were mainly women (63, 83%), had a mean BMI of 42.5, and predominantly classified their ethnicity as 'white Australian' (n = 61 80%). Additional file 2 provides a selection of narratives to illustrate each of the following categories.

### What motivates people living with obesity to diet?

All participants had attempted to lose weight numerous times in their lives, and in general had attempted to diet from their early teens. Whilst weight loss was the underlying motivating factor for all participants, participants gave a number of associated motivating factors. These included overall health and wellbeing (n = 16, 21%); preparation for lap banding surgery (n = 3, 4%); increased mobility (n = 6, 8%); not wanting to die (n = 4, 5%); advice from a health professional (n = 11, 14%); wanting to participate more fully in their children's lives (n = 7, 9%); wanting to participate in social activities (n = 11, 14%); not wanting to be ridiculed about their weight (n = 4, 5%); wanting to be socially accepted (n = 16, 21%); and wanting to establish a long term romantic relationship (n = 3, 4%).

### Weight loss techniques

The most popular weight loss techniques were *Weight Watchers *(n = 53, 70%); pharmaceutical medications – including Orlistat (n = 19, 25%) and Phentermine (21, 27%); complementary medicines (n = 37, 48%); Jenny Craig (n = 35, 46%); slimming milkshakes (n = 31, 40%), and periods of 'starvation' (n = 20, 26%). Diets and weight loss solutions mentioned by participants are shown in Additional file 3. There was an interesting line of progression in patterns of dieting. Participants stated that they had started with 'fad' diets published in magazines as teenagers (n = 37, 49%), before progressing to more formal commercialised diets. Most commonly the first commercial diet tried was *Weight Watchers*, followed by *Jenny Craig*. When these failed, some participants stated that they then tried more extreme techniques, such as pharmaceutical medications, and very low calorie diet supplements (such as slimming shakes). There was considerable overlap between the interventions with participants trying multiple weight loss strategies – sometimes concurrently (n = 19, 25%). These participants most commonly used dieting in conjunction with pharmaceutical medications.

The majority of participants were initially introduced to a particular diet by a member of their social network, such as a family member (n = 27, 36%), a friend (n = 13, 17%), or workmate (n = 11, 14%). Many (n = 37, 49%) were able to relate a story of a sister, mother or friend who has lost large amounts of weight on a commercial diet – in particular *Weight Watchers*. Some (n = 23, 30%) stated that the diet success stories of people within their networks gave them hope that the diet would work for them. Members of social networks played a vital role in encouraging participants to try different types of diets. Many described diet groups as a social activity that they went to with their family members or friends. This was particularly true of mothers and daughters, spouses and partners, and sisters. Others spoke of their diet group as a place where they had a sense of belonging, had some social contact, and had a sense of solidarity with other overweight and obese individuals. Given that these individuals also spoke of a sense of isolation, stigma and discrimination in wider society, the 'diet group' was perceived as a place of social acceptance. However, the diet group also reinforced low self-esteem, negative feelings, and self-blame, and was far from an accepting and supportive space for individuals.

Participants were asked why they tried particular diets. Most participants who had joined *Weight Watchers *stated that they had joined because a friend or family member was currently "*doing Weight Watchers*". Furthermore, those who had joined *Weight Watchers *said that they had tried the diet on many different occasions with a friend or family member, again highlighting the desire of many participants to take part in a social activity with their peers. Unlike any other commercial diet participants spoke of a cycle of losing weight on the *Weight Watchers *diet, stopping the diet, gradually gaining weight, and returning to *Weight Watchers *again. Others (n = 31, 41%) stated that they enjoyed the supportive environment of *Weight Watchers*, the regular weigh ins, the "*sensible eating program*", the community based nature of the program, the social nature of the groups, taking part in an activity with their family members or friends, and that it was more affordable than many other dieting options. Participants were also more likely to state that they went back to *Weight Watchers *repeatedly over time (n = 22, 29%), suggesting that whilst on the surface the support was good, the 'diet' itself was unsustainable for most participants and did not promote long term change. A small minority of participants (n = 4, 5%) stated that *Weight Watchers *was inappropriate for their needs, that they had felt humiliated within the group setting, that there had been too much food, or that they had not lost weight on the plan. These individuals were more likely to attend the group alone, and spoke of feeling unsupported. Thus, the positive aspects of attending *Weight Watchers *may actually be linked to the positive effects of the social network, rather than the program itself.

Participants who had tried pharmaceutical medications were generally happy with the amount of weight they lost on the medications, but were unhappy with the side effects of the medications. This was particularly true of participants' experiences of *Phentermine*. Participants who had tried *Orlistat *were also dissatisfied with the cost of the drug, and found it difficult to afford over a long period of time.

Participants' accounts of their experiences of *Jenny Craig *were much different. The vast majority of individuals who tried *Jenny Craig*, did so alone. Those who had joined *Jenny Craig *spoke of being *"seduced" *by the *"spiel" *given by the consultants. Some (n = 13, 17%) stated that the expense of *Jenny Craig*, and the amount of time they would need to be on the program to lose a substantial amount of weight was unrealistic. Most of those who had tried a *Jenny Craig *program stated they had only done so once (n = 14, 18%). Apart from the relationship with their consultant, many felt that they were *"alone" *or isolated when they were on the *Jenny Craig *diet.

Of the seven participants who had tried *Optifast*, most commented that they were dissatisfied that there was no other advice given to them about lifestyle change.

### Short and long term effects of weight loss techniques

In the short term, the vast majority of participants stated that they had lost weight when they had dieted. Over half of participants stated that they had had short-term success with dieting. Many were able to identify the time when they had lost the most weight, which diet had 'worked', and how much they had lost. Participants described the marked physical and emotional difference that losing weight had had on their lives. Words such as *"euphoric"*, *"very happy"*, *"delighted" *and *"ecstatic" *were used to describe how some participants felt emotionally when they lost weight. Some women spoke in detail about becoming *"attractive to men" *and being able to form romantic relationships when they lost weight. Other participants stated that when they lost weight they physically felt *"more comfortable"*, that they could *"move more" *and that they could *"keep up with the kids"*.

However, for the vast majority of participants, the euphoria associated with weight loss was short lived. Some participants (n = 16, 21%) stated that they felt like they were a *"failure" *because they had had friends or family members who had had success with commercial diets, when they had not. Others stated that not being able to maintain the weight loss had made them feel "*depressed*", "*angry*" or "*cross*".

### Why diets don't work: Is it the diet, or is it me?

Twenty participants commented that they were unable to maintain dieting because the diets themselves were *"unrealistic", "unsustainable"*, were *"too expensive", "didn't address my lifestyle", "focused on food rather than changing my behaviour"*, were *"boring" *or *"made me constantly think about my next meal"*. Some participants (n = 12) commented that they were also confused about which choices were correct, because of the different messages that were given by different diet companies. Many highlighted that diets were impossible to stick to, but were successful as long as you remained on them:*"They all work... when you stick to them. It's when you go off them that they don't." *One individual commented that she repeatedly gave up diets because *"sometimes you just want to be normal"*. This highlighted the sense of otherness participants felt in constantly restricting their lifestyles in their weight loss quest.

A third of participants (n = 25, 33%) blamed themselves or an associated life event for being unable to stick to or continue with a weight loss plan. Some stated that they had a personality type which expected a *"quick fix"*, and were dissatisfied when they did not see instant results, or if their weight fluctuated on programs. Other reasons for discontinuing with a diet included: emotional problems, stress, feeling physically unwell, moving to a new job, changes in financial circumstances, lack of willpower, Christmas, an inability to follow the diet *"strongly enough"*, or not being *"committed enough"*. This again reinforced that participants blamed themselves rather than the diet for their failure to lose weight.

Participants also commented on the pressure they felt from family and friends once they lost weight. Some (n = 15, 20%) commented that they became upset when people commented on or drew attention to the amount of weight they had lost because participants knew that they would probably put the weight back on again. As we have reported elsewhere, participants also commented that if they lost large amounts of weight, friends and family members would comment that they *"looked sick" *or *"too thin" *and would try to encourage them to stop dieting.[11] In some cases participants stated that they felt family members and friends would try to *"sabotage" *their attempts to lose weight. Dieting with a member of their social network was a double edged sword for participants. Some stated that they felt guilty if they lost more weight than their 'diet partner', whilst others felt disillusioned if they lost less weight or if they did not have the same amount of 'success' on the diet.

### Physical activity

Very few participants said that physical activity was a part of their weight loss strategy or was used in combination with dieting. When we asked participants if they had tried exercising there seemed to be even more barriers in their way than when we spoke about dieting. The majority of participants (n = 63, 83%) said that they found exercising difficult because of their weight, physical health problems, that they could not afford gym subscriptions, or personal trainers, did not have time to exercise, or felt uncomfortable or embarrassed about taking part in organised exercise activities. Other reasons for not exercising included, *"it is dark when I get home from work, so I can't go for a walk"*, *"feeling fat"*, *"too lazy" *and *"I can't be bothered"*. Participants stated that it was very difficult to exercise on their own, and wanted an intervention in which someone else would take the responsibility for motivating them and helping them to start being more physically active. *"Give me a personal trainer that gets me out of bed every morning and makes me exercise, and yeah, I'd lose weight." *Just as with commercial diets, it was interesting that contemplating an activity by themselves was difficult for participants, and again highlighted the need to look for solutions which positively involved informal and formal networks of support.

Participants also spoke of being emotionally humiliated, embarrassed or daunted when they attempted to exercise alone. Swimming and walking were the two most common forms of exercise recommended to participants by health professionals. Whilst many participants felt that swimming would be the ideal exercise for them, most said that they felt ashamed and embarrassed at going to a place where they would have to bare so much of their body to others. One participant described how after her GP recommended that she try swimming, she wore long track pants and a sweatshirt in the water so that other swimmers would not see her body. Another woman commented that when her dietician recommended walking, she walked the local streets at 5 am so that no-one else would see her. Again, the sense of isolation in these narratives was a strong contrast to those in which participants spoke positively about being involved in activities with their network members.

### What would work?

Most participants (n = 53, 70%) were quick to point out that there was no magic diet that could help people with obesity. Rather they were very keen to explain, with many examples, that different programs and support systems would be helpful to different people, and that each individual was different. Many highlighted the difficulty of trying to lose weight by themselves and without support. Participants were critical that many diets were extremely expensive, and out of reach of those who had large amounts of weights to lose (n = 17, 22%). However, nearly two-thirds (n = 49, 64%) thought that 'dieting' was an effective way to lose weight. When asked what obese people needed to do to lose weight, 80% of participants stated that they needed to diet. Very few participants mentioned exercise or physical activity as part of a comprehensive weight loss strategy.

There was an underlying theme that participants wanted someone else to take responsibility for helping them to lose weight. Many felt that primary care providers, such as General Practitioners (GPs) were the best people to help them do this. Whilst they had tried to lose weight on many occasions, the majority now felt that they were unable to do so by themselves. This may also explain why a number of participants reluctantly felt that obesity surgery was the only option available to them.

Some participants stated that the best solutions for people living with obesity were those which would be accessible, affordable, long term, and engage them in developing personalised plans which would work for them. Many spoke of the need to empower individuals through supportive programs with other overweight individuals, to engage them in making healthy lifestyle choices, and in living happy, healthy lives. Some believed that the stigma associated with obesity made seeking help extremely difficult, and that there should also be programs focused on dispelling the myths that people living with obesity were lazy and unmotivated, and to blame for their weight gain. So while most participants stated that diets were an effective way to lose weight, the vast majority stated that interventions should not focus on weight loss, but overall lifestyle changes.

## Discussion

Participants in this study predominantly turned to dieting rather than 'lifestyle changes' or exercise to help in their efforts to lose weight. Three interesting explanations emerged from this paper.

### 1) People living with obesity have been 'socially conditioned' to turn to diets for a cure for their obesity, and to blame themselves when diets fail

For most of their lives, starting in early childhood, participants were told that if they dieted they would lose weight. Diets are marketed as magical, quick fix solutions to excessive weight. Research has shown that frequent exposure to messages about weight loss and dieting may strongly influence weight control behaviours [15]. This was true for participants in this sample who reported engaging in repeated and often extreme attempts to lose weight from an early age.

Whilst loaded with unrealistic goals and expectations, most diets did lead to some form of weight loss, at least in the short term. However, participants were unable to sustain weight loss, and felt that they were to blame when the diet failed [16,17]. Participants had also spent much of their lives trying to find a diet that would work. This was also evident in participants' recommendations for the need for many different types of personal plans to suit each individual. Participants also had unrealistic expectations about what they could achieve through dieting alone, in many cases turning to the diets or interventions such as obesity surgery which they perceived would lead to the greatest weight loss in the least amount of time. This finding concurs with others who have hypothesized that dieters have unrealistic expectations about the process and likely success of dieting [18].

### 2) It is difficult for people living with obesity to engage in exercise or physical activity

It was very clear from the results that people living with obesity are reluctant or find it extremely difficult to engage in physical activity. Why is this? Is it that diets are perceived to be a far easier solution than exercise programs? There is after all, a big difference between the amount of emotional and physical effort required to 'go on a diet' (very low initially) and the amount of emotional and physical effort required to increase physical activity (usually significant). Exercise is not sold as a magic solution to weight loss. In fact the messages about exercise are that it is very hard – i.e. the no pain no gain paradigm. Whilst some participants felt that they were physically incapable of exercising because of health difficulties, many also felt emotionally uncomfortable or publicly humiliated when trying to engage in the types of exercised recommended to them by physicians. The sense of isolation in taking part in physical activity was clear in most participants' narratives. Many participants felt disempowered, particularly in relation to physical activity. Public health workers and researchers should explore whether community based participatory strategies from other successful public health interventions, are adaptable and transferable to obesity prevention and health promotion [19,20] and how we may help in engaging and supporting people in making these lifestyle changes [21]. This is particularly important given emerging evidence that suggests that overweight individuals who are also physically active can significantly reduce their risks of coronary heart disease [22]. and that those who are 'fat' and 'fit' have lower risks of mortality than those individuals who are normal weight but inactive [23].

### 3) Social networks have both a positive and negative effect on efforts to lead more healthy lifestyles

Participants were strongly influenced by the attitudes and opinions of their informal social network members – especially family members. Whilst social networks were key in encouraging participants to try different diets, they were also instrumental in disrupting participants' weight loss attempts. Participants also sought out semi-formal social networks they could identify with, such as *Weight Watchers*, which provided a supportive environment, but also did not lead to any long-term change. It is well acknowledged that both formal and informal social networks have been shown to be a very powerful tool in encouraging positive health behaviour, social support, self-esteem, identities and perceptions of control [24]. Self-management and peer education strategies have also been shown to be highly effective motivators in addressing chronic illness [25]. Further investigation into how positive aspects of social networks may be utilised in obesity prevention and health promotion strategies is needed.

## Conclusion

We are very good at understanding the direct causes of health problems in obesity, but we are less successful in recognizing and acting on more upstream determinants such as individual's lack of empowerment, the impact of social networks, and the impact of the broader messages individuals receive about how to achieve healthy lifestyles. This may be largely due to the absence of the voices of those living with obesity in obesity research or in the planning of public health interventions. Whilst many individuals classified as obese receive numerous instructions about what to do to address their weight, very few are given appropriate long-term guidance or support. Public health approaches to obesity must engage and genuinely involve those living with obesity in the design, trial and evaluation of interventions if patterns of social change are to occur.

## Competing interests

The authors declare that they have no competing interests.

## Authors' contributions

ST was the lead investigator on the study (with PK). She was involved in the design of the study, conducting interviews, analysing data, and writing the manuscript. JH was involved in the design of the study, analysing data, and critically revising the manuscript. AK was involved in the design of the study, conducting interviews, analysing data, and critically revising the manuscript. RK was involved in the interpretation of data and in critically revising the intellectual content of the manuscript. PK was the lead investigator on the study (with ST). He was involved in the design of the study, conducting interviews, analysing data, and critically revising the manuscript.

## Supplementary Material

Additional file 1 table one. Participant characteristics. Click here for file

Additional file 2 diet paper box one. Participant narratives. Click here for file

Additional file 3 diet paper box two. Weight loss interventions tried by participants. Click here for file
