# Supplementary material for: "They all work...when you stick to them": A qualitative investigation of dieting, weight loss, and physical exercise, in obese individuals
Source: Nutr J. 2008 Nov 24;7:34. doi: 10.1186/1475-2891-7-34 (PMC2607302; doi:10.1186/1475-2891-7-34)
Supplement: Additional file 1 — table one. Participant characteristics. [file 1475-2891-7-34-S1.doc]

**Table One**

**Participants Characteristics**

| **Characteristic** |  | **Characteristic** | **N (%)** |
| --- | --- | --- | --- |
| **Age**  Range  Mean | 16-72  47 | **Gender**  Female | 63 (83%) |
| **Marital Status**  Single  Married  Divorced  Widowed | 27 (36%)  31 (41%)  12 (16%)  6 (8%) | **Education**  At least completed High School | 34 (45%) |
| **Geographical Location**  Rural | 28 (37%) | **Employment Status**  Unemployed | 39 (51%) |
| **Weight (kgs)***  Range  Mean | 72 – 225  119 | **BMI***  Range  Mean | 30-72.1  42.5 |
| **Obesity Classification***  Obese  Morbidly Obese | 32 (42%)  43 (57%) | Ethnicity White Australian  English  Greek  Italian  Lebanese  Irish | 61 (80%)  4 (5%)  3 (4%)  3 (4%)  3 (4%)  2 (3%) |

*One missing as not willing to report weight and height
