# Supplementary material for: "They all work...when you stick to them": A qualitative investigation of dieting, weight loss, and physical exercise, in obese individuals
Source: Nutr J. 2008 Nov 24;7:34. doi: 10.1186/1475-2891-7-34 (PMC2607302; doi:10.1186/1475-2891-7-34)
Supplement: Additional file 2 — diet paper box one. Participant narratives. [file 1475-2891-7-34-S2.doc]

**Box One**

**Participants Narratives**

*Motivations for Dieting*

“I want to look nice. I want to be like most of the other slimmer people. I don’t want to be the big fat blob.” Participant 077

“When I get uncomfortable I think ‘oh I want to lose some weight’. I’ve developed this huge roll under my breast. If I bend over to do my shoes there’s this big roll there. You sit down and it’s there. It pokes out. It’s very uncomfortable.” Participant 009

“I've gone to a carbohydrate modified diet. I really am sticking to the diet to get this weight off so I can fit into a skirt that I want to wear.” Participant 082

*Weight Loss Strategies*

“I invested a small fortune in [Jenny Craig] and had no success because I was well and truly sucked in by the fabulous girl at the first interview. She motivated me. I was going to take on the world.” Participant 013

“[My Doctor] told me all about the Acomplia. The FDA [has not] approved it yet. But where there is a will there’s a way! I got on the internet and I found it, so I got that shipped here.” Participant 036

“I’ve tried diets over and over and over again. I could easily make my life about losing weight and if I did that I would, there would be results. I have no doubt about that.” Participant 018

“I am not going to use my weight as an excuse to stop myself from doing things because I get enough of that from society saying ‘no you can’t do it because you’re big’, ‘no you can’t be a sexual, beautiful woman and be 135 kilos’. Well yes I can and I am. There are days when I think to myself, ‘oh god, just go and get the banding, you’ll lose 40 kilos, you’ll feel better, and people will f****** leave you alone’. But then I won’t be able to eat and you know eating is one of the things that I actually enjoy.” Participant 057

*Why don’t diets work?*

“My friends say, ‘Oh you’re so beautiful, don’t change, you won’t look the same if you’re thin’. I’ve had my ex-partner say to me ‘Don’t lose weight because then you’ll think you’re too good and I won’t love you anymore’. So you get both messages. Some people want you to stay the same because if you change, if you do lose the weight then you become a different person.” Participant 057

“Weight Watchers had been the only time that I've ever had much success. I did it for a while and probably lost about 5 kilos and felt a lot better. But then I got sick to death of never being able to have a drink and never being able to go out and have a meal and all that sort of stuff.” Participant 074

“I got probably within 2-3 kilos of that and I had everybody around me panicking that I was too thin and not eating enough.” Participant 040

*Short and Long Term Effect of Dieting*

“Really a failure, and shamed, and you know, I got to the stage where I thought I’m too scared to go back because I will not have lost any weight. And the last few weeks of going, I would starve myself for a couple of days before I went just so that I wouldn’t be a loser. And then, if I didn’t go one week, I wouldn’t go back. And I’m not sure that that’s the diet, that’s the way to approach, for me.” Participant 049

[And how did you feel after you stopped it?] “Just as bad as before I started it.” Participant 008

*Physical Activity and Exercise*

“When you’re fat and feeling awful you just don’t want to get up and exercise. I’m just too lazy to get to the swimming pool. I’ve got 47 swims left on my Swim Ticket, and I just can’t even be bothered getting to the swimming pool door.” Participant 058

“I sometimes think the day is beautiful and I want to go with the pusher and walk and walk all day. But I don’t feel motivated. I think ’It’s a nice day, I will walk all day and then pick up the kids’. But I don’t go. I just don’t do it. I don’t know, for some reason I don’t feel very motivated to do it.” Participant 056

*What would work*

“I don’t want a quick fix. I don’t expect a quick fix. I’m a little bit past that. But I want something where I can feel human again, where I can feel like a person again, where I’m not constantly thinking ‘I’ve got to do this’ or ‘I’ve got to do that’.” Participant 011

“Let’s not focus on weight loss, let’s focus on health and lifestyle. Yes you can choose not to eat that piece of steak with the big piece of fat on it or you can choose to eat it. That’s your choice. You can choose to walk around the block three times a week or you can choose not to do it. That’s your choice too. So it has to be about empowering bigger people to make choices that will benefit their lifestyle, their life. It shouldn’t be about weight loss, let’s take it away from the kilos and the pounds and you’ve got to lose, lose, lose.” Participant 003
