# Supplementary material for: "They all work...when you stick to them": A qualitative investigation of dieting, weight loss, and physical exercise, in obese individuals
Source: Nutr J. 2008 Nov 24;7:34. doi: 10.1186/1475-2891-7-34 (PMC2607302; doi:10.1186/1475-2891-7-34)
Supplement: Additional file 3 — diet paper box two. Weight loss interventions tried by participants. [file 1475-2891-7-34-S3.doc]

**Box Two**

**Weight Loss Interventions Tried by Participants**

##### Diets/Diet Supplements

Weight Watchers Jenny Craig

Atkins Diet Sureslim

Nutri System MediTrim

OPTIFAST Lite and Easy

Soup Diet Grapefruit Diet

CSIRO Diet Gloria Marshall

T.O.W.N (Take Off Weight Now) Tony Ferguson Slimshakes

Slimfast Hip and Thigh

Potato Diet Zone Diet

Low GI Beverly Hills diet

Modifast Water and Celery Diet

Thin over Mind Diet Israeli Diet

Bikini Diet Demos Russos Diet

Richard Symmonds Diet Israeli Army Diet

Stop the Aitkin’s Revolution Diet No eating after 7pm Diet

Montiac program The Pritikin program,

The Fit for Life Diet Modified Carbohydrate Diet

Swedish Milk Diet Apple and Onion Diet

##### Other Interventions

*Pharmaceutical*

Xenical (Orlistat) Duramine

Zoloft **Sibutramine**

Accomplia Reductol

Tenuate Dospan

*Surgery*

Lap Banding Stomach Stapling

Liposuction

*Complementary or Alternative Therapies*

Acupuncture Hypnotherapy

Massage Herbalist

Chinese Medicine Tai Chi

Osteopath Holistic Therapist

Naturopath Ear Stapling

Electric stimulation Ford Pills

Herbal Teas

*Support Groups*

Overeaters Anonymous Weight Loss Support Group

*Physical Activity*

Walking Swimming

Gym membership
